# Developmental and Ultrastructural Characterization of *Trypanosoma theileri*-like Flagellates in a Horsefly *Hybomitra montana*

**DOI:** 10.3390/pathogens15070668

**Published:** 2026-06-25

**Authors:** Alexander O. Frolov, Anna I. Solovyeva, Marina N. Malysheva, Maria E. Belokon, Grigory N. Machakhtyrov, Varvara A. Machakhtyrova, Anatoly A. Bondarev, Maria S. Maximova, Anna I. Ganyukova

**Affiliations:** 1 Zoological Institute of the Russian Academy of Sciences, 199034 St. Petersburg, Russia; frolal@yandex.ru (A.O.F.); malmarnik@yandex.ru (M.N.M.); mirrobella@mail.ru (M.E.B.); mariamaximovabio@gmail.com (M.S.M.); anna.ganyukova@gmail.com (A.I.G.); 2 Institute of Cytology of the Russian Academy of Sciences, 194064 St. Petersburg, Russia; 3 Academy of Sciences of the Republic of Sakha (Yakutia), 677007 Yakutsk, Russia; aylga@mail.ru (G.N.M.); varvara-an@mail.ru (V.A.M.); anatolij.bondarev.9841@mail.ru (A.A.B.)

**Keywords:** cytostome–cytopharynx complex, *T. theileri*-like trypanosomes, life cycle, vector

## Abstract

The subgenus *Megatrypanum* Hoare, 1964, with the type species *Trypanosoma theileri* Laveran, 1902, comprises stercorarian trypanosomes of mammals. A substantial portion of this subgenus consists of *T. theileri*-like trypanosomes parasitizing cervids and bovids worldwide. Similar to most other members of the genus *Trypanosoma* that lack obvious economic importance, the biology of *T. theileri*-like trypanosomes remains poorly understood. In particular, fundamental aspects such as their host specificity, host–parasite interactions, and the morphology of developmental stages have been studied only to a limited extent. In this work, we provide a detailed description of the development and cellular organization of *T. theileri*-like trypanosomes in the horsefly *Hybomitra montana* using transmission electron microscopy (TEM). We show for the first time that *T. theileri*-like trypanosomes possess a well-developed cytostome–cytopharyngeal complex, morphologically similar to those in other stercorarian trypanosomes. This complex is present in the studied trypanosomes at the epimastigote stage and degrades during metacyclogenesis. In the host ileum, epimastigotes and trypomastigotes at different stages of metacyclogenesis are embedded in a fibrillar matrix that isolates them from the gut lumen. This promotes their accumulation in the vector, thereby increasing the efficiency of future infection of the vertebrate host, which occurs via contamination of the oral mucosa.

## 1. Introduction

The genus *Trypanosoma* Gruby, 1843, comprises dixenous (two-host) parasitic flagellates of the family Trypanosomatidae (Kinetoplastea), which parasitize the blood of vertebrates and utilize various blood-sucking invertebrates as vectors. Trypanosomes are one of the most important groups of parasitic protists, including several major pathogens responsible for zoonotic infections in humans and animals, such as *Trypanosoma brucei* (sleeping sickness) and *Trypanosoma cruzi* (Chagas disease) [1].

*Trypanosoma theileri* Laveran, 1902, was among the first representatives of the genus Trypanosoma found in mammals [1]. Initially discovered in the blood of cattle in Africa, these trypanosomes were later recorded in ruminants worldwide. The pathogenic potential of *T. theileri* has not yet been fully clarified (see Section 4).

*T. theileri* was designated by Hoare as the type species of the subgenus *Megatrypanum* Hoare, 1964. The primary vectors of *T. theileri* in cattle are horse flies (Tabanidae), and infection of vertebrate hosts occurs via contamination of the oral mucosa [2]. The subgenus *Megatrypanum* further includes *T. melophagium* Flu, 1908, a parasite of sheep blood transmitted by the sheep ked (*Melophagus ovinus*), and *T. theodori* Hoare, 1931, a parasite of goat blood transmitted by the goat ked (*Lipoptena capreoli*) [1]. Subsequently, the subgenus was expanded to include trypanosomes from cervids: the European and North American *T. cervi* Kingston and Morton, 1975, and *T. trinaperronei* Teixeira, Camargo, and García, 2020, described in the Americas [3]. Their vectors are the deer keds *Lipoptena cervi* and *L. mazamae*, respectively. Traditionally, all these parasites are collectively referred to as *T. theileri*-like trypanosomes. Recent studies have identified at least three phylogenetic groups (TthI–III) within the clade uniting *T. theileri*-like trypanosomes, each represented by numerous genotypes. Furthermore, group TthII includes all three species of these trypanosomes described to date [3,4,5,6]. The main vectors of *T. theileri*-like trypanosomes of the TthI group are horse flies (Tabanidae), while those of the TthII and TthIII groups are transmitted by keds (Hippoboscidae), mosquitoes, sandflies, and potentially other blood-sucking Diptera [5]. The actual diversity and boundaries of the subgenus *Megatrypanum* remain insufficiently defined. Phylogenetic and phylogenomic studies demonstrate that the closest relatives of *Megatrypanum* are not other members of Hoare’s “Stercoraria” from the subgenera *Herpetosoma* and *Schizotrypanum* [1], but rather certain avian trypanosomes. These avian parasites form a distinct cluster within a clade composed predominantly of mammalian parasites and are paraphyletic relative to *T. theileri* [7,8].

The rapid development of electron microscopy in the second half of the 20th century provided new insights into the cellular diversity of eukaryotic microorganisms. However, in the genus *Trypanosoma*, this progress has primarily focused on a limited number of key representatives. Information on the cellular organization of most trypanosomes remains fragmentary and is derived primarily from taxonomic descriptions of individual species or studies on host–parasite interactions. Given that current estimates place the number of species in the genus *Trypanosoma* at approximately 500 [7], any comprehensive comparative analysis of their ultrastructural organization is currently difficult, if not impossible. To date, understanding of the cellular organization of *T. theileri*-like trypanosomes has been limited to sparse data on the development of *T. melophagium* (TthII) in the sheep ked *Melophagus ovinus* [9,10] and in culture [11], alongside similar studies of *T. theileri*-like trypanosomes from the TthI group [6,12].

In this study, we investigated the ultrastructure and host–parasite interactions of *T. theileri*-like trypanosomes from the TthI group (genotype Tthα) in the horse fly *Hybomitra montana* (Tabanidae) from Yakutia. This genotype was also recently detected in the horse flies *Hybomitra muehlfeldi* and *Chrysops divaricatus* (isolates KrSl7 and KrSl4, respectively) in Northwestern Russia [6,13]. Using TEM, we demonstrate for the first time the presence of a well-developed cytostome–cytopharyngeal complex in *T. theileri*-like epimastigotes, which opens onto the cell surface and is spatially separated from the flagellar pocket opening. We provide a detailed description of the main cellular compartments of trypanosomes of the Tthα genotype in vivo and demonstrate that the organization of several organelles undergoes substantial changes both during metacyclogenesis and during adaptation to in vitro culture conditions. We also describe the development of trypanosomes in the ileum of the horse fly and propose a hypothesis explaining the mechanism underlying parasite accumulation in this part of the host gut.

## 2. Materials and Methods

### 2.1. Material Collection

Horse flies (*Hybomitra montana*) were collected using an entomological net on 12 July 2024, in the vicinity of Verkhoyansk, Republic of Sakha (Yakutia) (67.536369 N, 133.394925 E). After capture, the insects were euthanized with chloroform and dissected in normal saline solution. The insect ileum was isolated and divided into three parts for preparation of smears, DNA extraction, and fixation for TEM.

As we had previously performed a detailed morphological study of cultured *T. theileri*-like trypanosomes of the KrSl7 isolate [12], which belongs to the Tthα genotype, we selected sample Gya-24143 from the Yakutian horse fly *H. montana*, also infected with *T. theileri*-like trypanosomes (Tthα genotype), to obtain comparable data for this work.

### 2.2. Light Microscopy

Smears prepared from the contents of the ileum fragment were air-dried and fixed in 96% ethanol for 30 min, then stained with Romanowsky-Giemsa for 15–20 min (pH 6.8). Micrographs were obtained using a Leica DM 2500 microscope (Leica Microsystems GmbH, Wetzlar, Germany) equipped with a UCMOS14000KPA 14-Mpx digital camera (ToupTek, Hangzhou, China).

### 2.3. Transmission Electron Microscopy and Statistical Analysis

For transmission electron microscopy (TEM), a fragment of infected ileum was fixed in 1.5% glutaraldehyde in 0.1 M cacodylate buffer for 1 h at 0 °C. After washing in 0.1 M cacodylate buffer containing 5% sucrose, the sample was post-fixed in 2% OsO_4_ in 0.1 M cacodylate buffer. Following dehydration in a graded series of ethanol and propylene oxide, the ileum pieces were embedded in Epon-Araldite resin. Ultrathin sections (60 nm thick) were prepared using a Leica UC-6 ultramicrotome and contrasted with a saturated aqueous solution of uranyl acetate (1 h) and Reynolds’ lead citrate [14] (5 min). The sections were examined under a Morgagni 268-D microscope (FEI Company, Hillsboro, OR, USA) with an accelerating voltage of 80 kV. Organelle measurements were performed using UTHSCSA ImageTool for Windows, Version 3.0 (University of Texas Health Science Center, San Antonio, TX, USA). Comparisons of morphometric parameters of the same organelles between different developmental stages of Tthα trypanosomes were performed using the Mann–Whitney test in Statistica v.10 (StatSoft, Inc., Tulsa, OK, USA).

### 2.4. DNA Extraction, Amplification, and Sequencing

Genomic DNA was extracted from the infected ileum fragments of *H. montana* using the HiPure Universal DNA Kit (Magen, Guangzhou, China) according to the manufacturer’s protocol. The ~800-bp fragment of the 18S rRNA gene (encompassing variable regions V8, V3, V4, and V9) was amplified using the primer pair 1127F and 1958R [15]. A ~900-bp fragment of the glycosomal glyceraldehyde-3-phosphate dehydrogenase (gGAPDH) gene was amplified using the primer pair G3 and G4a [16]. The same primer pairs were used for sequencing. The resulting sequences of the sample Gya-24143 were deposited in GenBank under accession numbers PZ488411 (gGAPDH) and PZ484268 (18S rRNA gene fragment).

To determine the host species (*Hybomitra montana*), a fragment of the COI gene was amplified using the primer pair LCO1490 and HCOI2198 [17]. Insect identification was performed using the Barcode of Life Data System (BOLD; http://www.boldsystems.org). We were able to identify *H. montana* with high accuracy; all our samples showed >99% identity to the sequences in the database.

## 3. Results

A total of 16 female horse flies (*H. montana*) were collected and subsequently dissected in the vicinity of Verkhoyansk, Republic of Sakha (Yakutia). All individuals were found to be infected with trypanosomatids. Comparison of the 18S rRNA and gGAPDH gene sequences obtained from the infected insects with those available in GenBank revealed 14 cases of infection with Tthα trypanosomes (clade TthI) and two mixed infections involving both Tthα and Tthβ (clade TthII).

Sample Gya-24143 from an *H. montana* specimen infected with Tthα trypanosomes was selected for further ultrastructural analysis. A BLAST (https://blast.ncbi.nlm.nih.gov/Blast.cgi, last accessed 22 June 2026) search against GenBank revealed 100% identity of Gya-24143 to a 790-bp fragment of the 18S rRNA gene and 99.87% identity to a 765-bp fragment of the gGAPDH gene from trypanosomes previously identified in Northwestern Russia (isolate KrSl4). The single mismatch in the gGAPDH gene is attributable to a single ambiguous position in the KrSl4 sequence deposited in GenBank.

### 3.1. Host–Parasite Relationships

**Figure 1:**
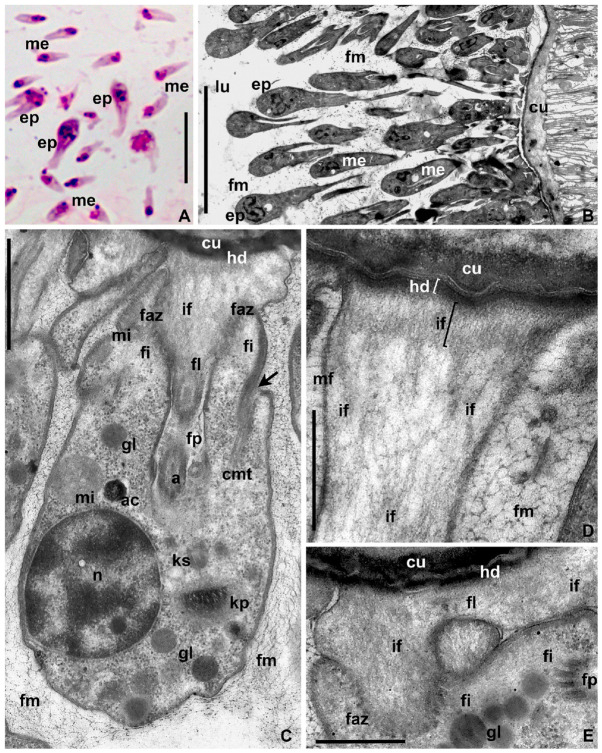
*T. theileri*-like Tthα trypanosomes in the ileum of the horse fly *Hybomitra montana*. (**A**)—light microscopy, Giemsa staining; (**B**–**E**)—TEM. (**A**)—epimastigotes and metacyclic trypomastigotes on smears from the horse fly ileum; (**B**)—*T. theileri* epimastigotes and metacyclic trypomastigotes embedded in the filamentous matrix forming a multilayered palisade on the cuticular lining of the horse fly ileum; (**C**)—*T. theileri* epimastigote attached to the ileum epicuticle surface by a shortened flagellum; (**D**)—the tip of an epimastigote flagellum attached to the horse fly ileum epicuticle; (**E**)—intraflagellar and cytoplasmic filaments contacting the FAZ of epimastigotes. Abbreviations: a—flagellar axoneme; ac—acidocalcisome; cmt—cytostomal microtubule group; cu—cuticular lining of the ileum; faz—flagellar attachment zone; fi—cytoplasmic filaments; fl—flagellum; fm—filamentous matrix; fp—flagellar pocket; gl—glycosomes; hd—hemidesmosome; if—intraflagellar filaments; kp—kinetoplast; ks—kinetosome; lu—ileum lumen; me—metacyclic trypomastigotes; mf—flagellar membrane; mi—mitochondrion; n—nucleus. The arrow indicates the cytostome opening. Scale bars: (**A**)—10 μm; (**B**)—5 μm; (**C**)—1 μm; (**D**,**E**)—0.5 μm.

**Figure 2:**
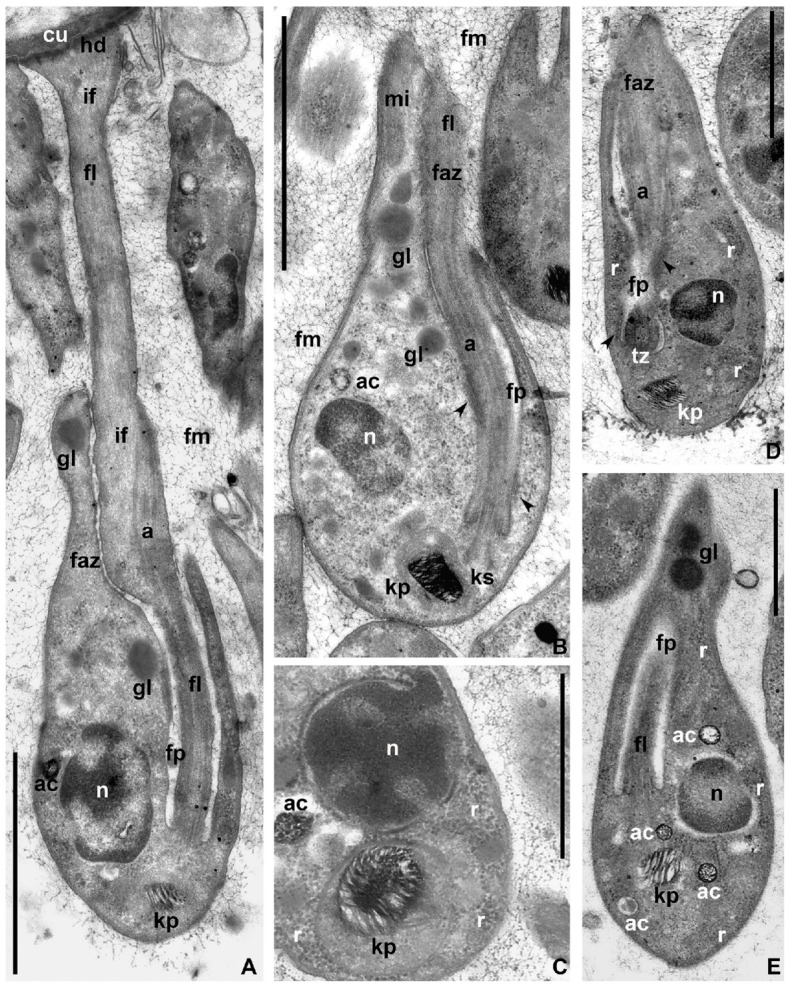
Ultrastructure of trypomastigotes in the ileum of the horse fly *Hybomitra montana*. (**A**)—trypomastigote attached to the host ileum cuticle by an expanded flagellar tip; (**B**)—unattached trypomastigote with translucent cytoplasm; (**C**–**E**)—metacyclic trypomastigotes. Abbreviations: a—flagellar axoneme; ac—acidocalcisome; cu—cuticular lining of the ileum; faz—flagellar attachment zone; fl—flagellum; fm—filamentous matrix; fp—flagellar pocket; gl—glycosomes; hd—hemidesmosome; if—intraflagellar filaments; kp—kinetoplast; ks—kinetosome; mi—mitochondrion; n—nucleus; tz—flagellar transition zone; r—ribosomes. Arrowheads indicate microtubules at the flagellar pocket wall. Scale bars: (**A**,**B**)—2 μm; (**C**–**E**)—1 μm.

In the ileum of *H. montana*, the Tthα flagellate population comprises epimastigotes (Figure 1A,C) and trypomastigotes (Figure 1A and Figure 2A), which attach to the cuticular lining of the gut via their flagella, as well as non-flagellated metacyclic trypanosomes (Figure 1A,B and Figure 2D,E). Cells of all types are intermingled. They are oriented with their anterior ends toward the gut wall, forming a multilayered “palisade” at the surface of the host enterocyte cuticle (Figure 1B). Regardless of the presence or absence of an external portion of the flagellum, the parasite cells do not leave the palisade and do not enter the ileum lumen. This is facilitated by a filamentous matrix in which they are embedded (Figure 1B,C and Figure 2A–E). The filaments forming this matrix, approximately 4–6 nm in diameter and arranged in multiple directions, create a three-dimensional network. By interacting with each other and with the surface of trypanosome cells, they form a “sheath” that isolates the parasites from the gut lumen.

Parasites surrounded by the filamentous matrix are able to attach to the surface of the ileum epicuticle using their flagella. Flagellates located in close proximity to the host ileum wall possess shortened flagella, in which the entire extracellular portion is transformed into a hypertrophied attachment organelle (Figure 1C–E). In contrast, cells located at some distance from the ileum wall possess long flagella and attach to unoccupied areas of the cuticle via the expanded distal tip of the undulipodium (Figure 2A).

A zonal hemidesmosome, 35–45 nm thick, is formed beneath the flagellar plasmalemma at the site of contact with the host gut epicuticle (Figure 1C–E and Figure 2A). Filaments with a diameter of 4–6 nm are observed at the inner surface of the hemidesmosome. These filaments are oriented from the anterior toward the posterior end of the flagellum and initially lie closely apposed, forming a compact bundle (Figure 1D). At a distance of approximately 200 nm from the inner surface of the hemidesmosome, individual filaments and small groups begin to separate from this bundle. While generally maintaining their orientation, they may bend and interweave, forming a loose network. This filament network fills the entire intraflagellar matrix of the modified undulipodium, extending from the hemidesmosome to the level of the proximal boundary of the flagellar attachment zone (FAZ) within the flagellar pocket (Figure 1C,E and Figure 2A). At the level of the FAZ, filaments of similar diameter (4–6 nm) are also present in the cytoplasm beneath the plasmalemma of the flagellar pocket. These filaments are often organized into bundles extending deeper into the cytoplasm (Figure 1C,E).

### 3.2. Ultrastructure of T. theileri-like Trypanosome Developmental Stages in H. montana

#### 3.2.1. Epimastigotes

**Figure 3:**
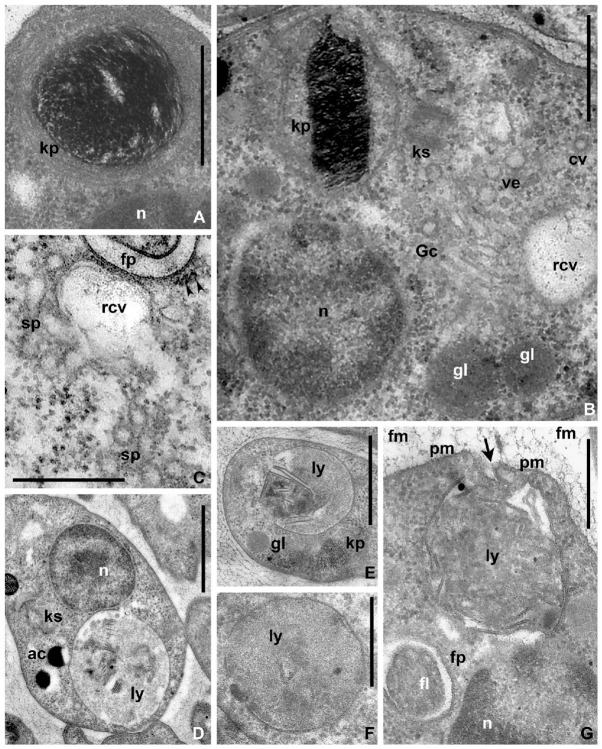
Ultrastructure of Tthα epimastigotes in the ileum of *Hybomitra montana.* (**A**)—transverse section through the epimastigote kinetoplast; (**B**)—cytoplasmic organization in the perinuclear zone of the epimastigote; (**C**)—contractile vacuole complex; (**D**–**G**)—giant lysosome-like vacuoles in the epimastigote cytoplasm. Abbreviations: ac—acidocalcisome; cv—coated vesicles; fl—flagellum; fm—filamentous matrix; fp—flagellar pocket; Gc—Golgi complex; gl—glycosomes; kp—kinetoplast; ks—kinetosome; ly—lysosome-like vacuoles; n—nucleus; pm—plasmalemma; rcv—contractile vacuole reservoir; sp—spongiome network; ve—vesicles of various origin. The arrow indicates perforation of the plasmalemma at the site of autophagosome content discharge; arrowheads indicate microtubules at the flagellar pocket wall. Scale bars: (**A**–**C**)—0.5 μm; (**D**)—1 μm; (**E**–**G**)—0.5 μm.

**Figure 4:**
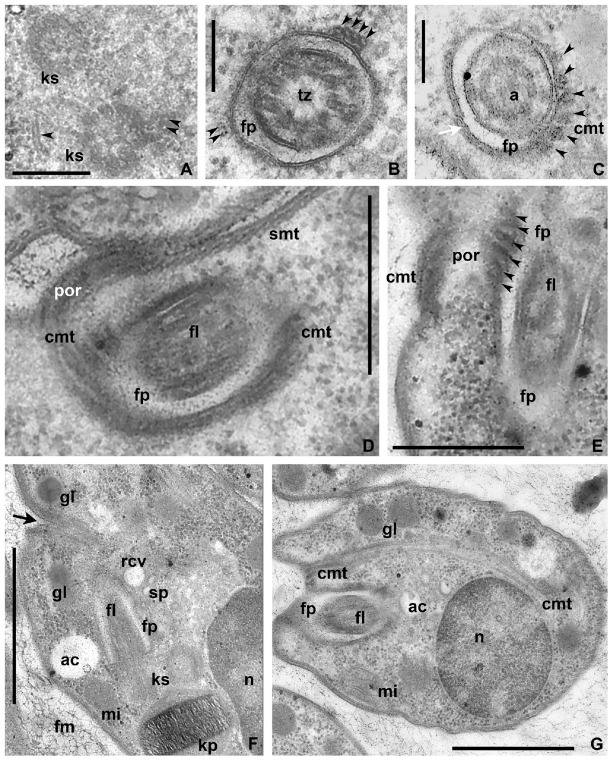
Ultrastructure of Tthα epimastigotes in the ileum of *Hybomitra montana* (continued). (**A**)—kinetosomes; (**B**,**C**)—transverse sections through the epimastigote flagellar pocket at different levels; (**D**,**E**)—preoral ridge; (**F**)—cytostome localization on the epimastigote cell surface; (**G**)—cytostomal microtubule group in the epimastigote cytoplasm. Abbreviations: a—flagellar axoneme; ac—acidocalcisome; cmt—cytostomal microtubule group; fl—flagellum; fm—filamentous matrix; fp—flagellar pocket; gl—glycosomes; kp—kinetoplast; ks—kinetosome; mi—mitochondrion; n—nucleus; por—preoral ridge; rcv—contractile vacuole reservoir; smt—submembrane microtubules; sp—spongiome network; tz—flagellar transition zone. The black arrow indicates the cytostome opening; the white arrow indicates the flagellar pocket collar zone; arrowheads indicate microtubules associated with the flagellar pocket. Scale bars: (**A**)—0.25 μm; (**B**,**C**)—0.2 μm; (**D**,**E**)—0.5 μm; (**F**,**G**)—1 μm.

**Figure 5:**
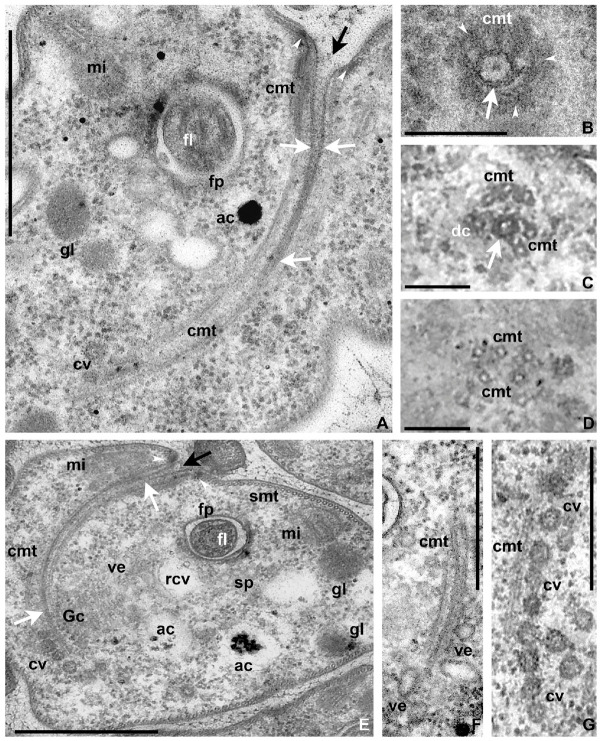
Organization of the cytostome–cytopharyngeal complex in Tthα epimastigotes in the ileum of *Hybomitra montana.* (**A**)—longitudinal section through the cytostome–cytopharyngeal complex; (**B**)—transverse section through the distal part of the cytopharyngeal channel at the boundary with the cytostome; (**C**)—transverse section of the middle part of the cytopharyngeal channel surrounded by cytostomal microtubules; (**D**)—transverse section through the cytostomal microtubules extending beyond the proximal end of the cytopharyngeal channel; (**E**)—cytostome–cytopharyngeal complex; (**F**)—uncoated vesicles associated with cytostomal microtubules; (**G**)—coated vesicles associated with cytostomal microtubules. Abbreviations: ac—acidocalcisome; cmt—cytostomal microtubule group; cv—coated vesicles; dc—cytostomal microtubule doublet; fl—flagellum; fp—flagellar pocket; Gc—Golgi complex; gl—glycosomes; mi—mitochondrion; rcv—contractile vacuole reservoir; smt—submembrane microtubules; ve—vesicles. The black arrow indicates the cytostome opening; white arrows indicate the cytopharynx; white arrowheads indicate the ridge of electron-dense material underlying the cytostome plasmalemma. Scale bars: (**A**)—1 μm; (**B**)—0.2 μm; (**C**,**D**)—0.15 μm; (**E**)—1 μm; (**F**,**G**)—0.5 μm.

Epimastigote cells are typically pear-shaped or nearly so (Figure 1A–C). Their flagellum, transformed into an attachment organelle, emerges from a flagellar pocket that opens either on the lateral surface of the cell or in a subterminal position (Figure 1C,E). In the latter case, a clear asymmetry in the position of the flagellar pocket opening relative to the longitudinal axis of the cell is maintained (Figure 1C). The nucleus is located in the central part of the epimastigote cell (Figure 1C, Figure 3B,D and Figure 4F). The mean diameter of interphase nuclei is 1.34 ± 0.05 µm (n = 17). A disk-shaped kinetoplast is located in close proximity to the nucleus, usually at the same level (Figure 3A,B). Kinetoplast DNA fibrils are tightly packed. The kinetoplast diameter (Figure 3A) is 0.575 ± 0.021 µm (n = 17), and its thickness (Figure 3B) is 0.331 ± 0.005 µm (n = 17). Occasional profiles of mitochondrial branches with lamellar cristae are observed mainly in the anterior part of the epimastigote cells (Figure 1C, Figure 4F,G and Figure 5E). Both kinetosomes are located between the anterior surface of the kinetoplast and the base of the flagellar pocket (Figure 1C and Figure 4A,F). The contractile vacuole complex includes a single large reservoir that either contacts the wall of the flagellar pocket (Figure 3C) or is located in its immediate vicinity (Figure 4F and Figure 5E). A system of vesicles and tubules 65–90 nm in diameter, forming a “spongiome”, is associated with the reservoir (Figure 3C and Figure 4F). The Golgi apparatus is situated in the cytoplasm between the nucleus and the contractile vacuole complex (Figure 3B). Its trans-side faces the anterior end of the cell. The cytoplasm surrounding the dictyosome contains numerous vesicles (Figure 3). In contrast to the organelles described above, which form a compact group in the central region of the cell, two types of organelles—acidocalcisomes and glycosomes—are distributed more randomly throughout the cytoplasm. Acidocalcisomes are spherical (Figure 1C, Figure 3D, Figure 4F,G and Figure 5A,E) with an average diameter of 0.23 ± 0.04 µm (n = 19). Glycosomes (Figure 1C,E, Figure 3B, Figure 4F,G and Figure 5A,E) are also spherical and have similar dimensions (0.24 ± 0.02 µm; n = 19).

Large lysosome-like vacuoles are present in the cytoplasm of many *T. theileri* epimastigotes (Figure 3D–G). Their mean diameter is 0.744 ± 0.047 µm (n = 25). The contents of these organelles are heterogeneous and include membrane fragments and various vesicles. Most of the vacuolar volume is occupied by a fine-grained matrix of moderate electron density (Figure 3D–G). In several cases, contacts between the membranes of these vacuoles and the epimastigote plasmalemma were observed, accompanied by the release of their contents outside the cell (Figure 3G). These lysosome-like vacuoles are primarily located in the post-nuclear region of the cytoplasm. The flagellar pocket of epimastigotes is relatively short and cylindrical (Figure 1C and Figure 4B,C). On the cytoplasmic side, the plasmalemma of the flagellar pocket is associated with two groups of microtubules (Figure 4B,C). Two microtubules originate in the electron-dense material at the basal part of the kinetosome (Figure 4A). They are oriented parallel to the longitudinal axis of the kinetosome and subsequently to the transition zone and flagellar axoneme, and can be traced up to the flagellar pocket collar (Figure 4B,C). The second group includes up to four microtubules. Near the kinetosome, they are oriented at an angle to its longitudinal axis (Figure 4A), then follow a helical trajectory around the flagellar pocket. At the level of the distal transition zone, they become aligned parallel to the longitudinal axis of the pocket, forming a compact “quartet” (Figure 4B). These microtubules also extend to the flagellar pocket collar. At the collar level, a group of six microtubules is observed (Figure 4C), hereafter referred to as cytostomal microtubules, as they are directly involved in supporting the cytostomal complex. Four of these six microtubules are surrounded by electron-dense material, whereas two remain free (Figure 4C). From the flagellar pocket collar, the cytostomal microtubules extend into the cytoplasm toward the cell periphery, where they bend by 180°, forming a characteristic “preoral ridge” (Figure 4D,E). Upon reaching the cytostome opening, they participate in the formation of the cytostome–cytopharyngeal complex (Figure 4F,G and Figure 5A–F). The cytostome opening is located on the lateral surface of epimastigote cells at a distance of 0.3–0.5 µm from the external opening of the flagellar pocket (Figure 1C and Figure 4F). The cytostome is an invagination of the plasmalemma, surrounded on the cytoplasmic side by a ridge of electron-dense material through which the cytostomal microtubules pass (Figure 5A,B,E). The mean external diameter of the cytostome opening is 183 ± 0.01 nm (n = 11). The plasmalemmal invagination continues into the cytoplasm as a relatively rigid cytopharyngeal channel, the diameter of which gradually decreases from ~80 nm distally to ~20 nm proximally (Figure 5A–C,E).

The length of the cytopharynx usually does not exceed 1 µm. It is oriented with its proximal end toward the nucleus and the Golgi apparatus (Figure 4G and Figure 5E). Cytostomal microtubules accompany the cytopharynx along its entire length and extend beyond its proximal end, reaching a total length of 2–2.5 µm, i.e., approximately twice the length of the cytopharyngeal channel. At the mid-level of the cytopharynx, up to seven microtubules are present, two of which typically form a doublet (Figure 5C). Six microtubules extend beyond the proximal end of the cytopharynx. Vesicles, including coated vesicles 70–90 nm in diameter, are frequently observed along these microtubules (Figure 5A,E–G).

#### 3.2.2. Trypomastigotes

The trypomastigote population in the horse fly ileum consists of flagellates at various stages of metacyclogenesis, leading to the formation of metacyclic trypanosomes. Trypomastigotes are easily recognizable in both light microscopy and TEM by the position of the kinetoplast, which is shifted beyond the posterior boundary of the nucleus (Figure 1A,B and Figure 2A–E). These stages are also characterized by a deep flagellar pocket (Figure 2A,B,D,E). A key ultrastructural difference between trypomastigotes and epimastigotes is the almost complete absence of transport system compartments in the former. We did not detect a Golgi apparatus, contractile vacuole complex (including spongiome channels), cytostome–cytopharynx, endoplasmic reticulum, or lysosomes in trypomastigote cells (Figure 2A–E). Only a single group of microtubules is associated with the elongated flagellar pocket, following a helical trajectory from the kinetosome toward the anterior end of the cell (Figure 2B,D).

The exact sequence of epimastigote transformation into metacyclic trypanosomes cannot be reliably reconstructed (i.e., described as a sequential progression of specific transitional forms) due to the asynchronous development of parasites in their natural population. However, the high frequency of certain forms suggests their non-random involvement in metacyclogenesis.

Based on these observations, several morphotypes can be distinguished within the trypomastigote population:

1. *Attached forms*. These are trypomastigotes that attach to the ileum cuticle via the expanded tips of their flagella, forming a zonal hemidesmosome (Figure 2A). The intraflagellar matrix of these forms is enriched in longitudinally oriented microfilaments (4–6 nm in diameter) and their bundles (8–17 nm in diameter). Notably, even when the free part of the undulipodium is relatively long, the flagellar axoneme does not extend beyond the level of the flagellar pocket, and the paraxial rod is not detected (Figure 2A). The cytoplasm of these trypomastigotes is relatively translucent and contains numerous ribosomes, acidocalcisomes, and glycosomes.

2. *Unattached trypomastigotes with translucent cytoplasm*. These forms are characterized by relatively light cytoplasm with numerous ribosomes and, frequently, an unusual orientation of the DNA-containing region of the kinetoplast, positioned at a significant angle to the longitudinal axis of the cell (Figure 2B). Their flagellum does not extend beyond the flagellar pocket, forming an extended FAZ with one of the pocket walls (Figure 2B).

3. *Metacyclic trypomastigotes*. These stages possess very dense cytoplasm. The kinetoplast disk occupies its typical position, perpendicular to the longitudinal axis of the cell (Figure 2D,E). In addition to the nucleus and kinetoplast, only occasional glycosomes and acidocalcisomes are present in the cytoplasm (Figure 2D,E). Notably, metacyclic forms almost completely lack free ribosomes; instead, ribosomes are mainly observed as small clusters located at the cell periphery (Figure 2D,E). The pronounced cell compaction observed during the formation of metacyclic stages is accompanied by the compaction of the major cellular organelles (Figure 2C). The nuclear diameter (0.93 ± 0.05 µm), as well as the thickness and diameter of the kinetoplast disk (0.33 ± 0.01 µm and 0.47 ± 0.02 µm, respectively) (n = 25), are significantly smaller (*p* < 0.002) than those of epimastigotes.

## 4. Discussion

*Trypanosoma theileri* Laveran, 1902, was described in the early 20th century from the blood of cattle in Transvaal (South Africa). Similar trypanosomes, often described under various names, were subsequently reported from ruminants worldwide [1]. All of these trypanosomes are characterized by large blood stages (40–100 µm), an elongated posterior end, and kinetoplasts positioned close to the nucleus. Owing to their morphological similarity and largely unknown life cycles, Hoare [1] grouped them within the subgenus *Megatrypanum* as “*Theileri*-like trypanosomes from ruminants”. Despite a long history of investigation and the wide distribution of *T. theileri*-like trypanosomes, very little is known about the biology of these parasites. They are regarded as non-pathogenic or opportunistically pathogenic trypanosomes, with infections typically remaining asymptomatic. In rare cases, particularly under conditions of co-infection or stress, infected animals may exhibit fever, anorexia, and anemia, and cases of abortion have also been reported [18,19,20,21,22,23]. Information on the vectors and host–parasite relationships of these trypanosomes also remains extremely limited [2,3,5,6,9,10,13,24,25,26,27,28]. Furthermore, in light of current knowledge regarding the genetic diversity of *T. theileri*-like trypanosomes [4,5,6], it should be acknowledged that much of the data obtained during the pre-molecular era are now difficult to verify.

In the present study, we focused on *T. theileri*-like trypanosomes from clade TthI (genotype Tthα) in the horse fly *Hybomitra montana* (Tabanidae). Although the vertebrate host for these trypanosomes has not yet been identified, available evidence suggests that Tthα trypanosomes parasitize cervids. In particular, their closest relatives are trypanosomes detected in the blood of sika deer *Cervus nippon* in Japan [6,29].

We analyzed the host–parasite relationships of Tthα trypanosomes in the ileum of the vector and investigated the cellular organization of the parasites at different stages of their morphogenesis.

### 4.1. Host–Parasite Relationships

Two distinct features characterize the development of *T. theileri*-like Tthα trypanosomes in the digestive system of horse flies: (i) the flagellates form a stable micropopulation only in one part of the host’s hindgut—the ileum; and (ii) motile stages with a free flagellum are either absent (this study) or extremely rare [6] in the host ileum. This significantly distinguishes the developmental strategy of Tthα trypanosomes in horse flies from that of *T. melophagium* (TthII) in sheep keds (*Melophagus ovinus*). In keds, *T. melophagium* colonizes all parts of the vector’s digestive system, from the midgut to the rectal ampulla [25]. Proliferation occurs in the midgut among epimastigotes with long free flagella, which anchor them to the epithelial brush border, and among pyriform epimastigotes that attach to the hindgut cuticle using shortened flagella [3,9,25]. Interestingly, these different developmental strategies do not appear to be linked to the phylogenetic lineage of *T. theileri*-like trypanosomes. It was previously established that the development of at least some TthII (genotype Tthβ) trypanosomes in horse flies follows the same scenario as Tthα (TthI) and, unlike *T. melophagium*, is also restricted to the vector’s ileum [6]. Conversely, the development of the avian trypanosome *T. corvi*, which is phylogenetically close to *T. theileri*-like species, in the hippoboscid *Ornithomyia avicularia* is identical to that of *T. melophagium* in *M. ovinus* [30]. This suggests that the type of invertebrate host (hippoboscids vs. tabanids) exerts a greater influence on the life cycle structure than the parasites’ phylogenetic position. Unfortunately, data on the development of other *T. theileri*-like species in keds and horse flies (including numerous cervid trypanosomes), as well as their relationships with other vectors (mosquitoes, biting midges, and blackflies), are absent in the literature, precluding a broader analysis of these patterns.

In the horse fly ileum, Tthα trypanosomes localize in close proximity to the cuticular lining of the intestinal epithelium, where their cells are embedded in a filamentous matrix. This matrix is believed to be secreted by the parasite cells themselves [6,31]. In *Leishmania*, a similar matrix is thought to consist of a mucin-like filamentous proteophosphoglycan, which forms a dense three-dimensional filament network with a cohesive function [32]. The formation of a filamentous matrix surrounding various trypanosomatids (not only *Trypanosoma* spp.) has been described in the cuticularized foregut and hindgut of many insects across different taxonomic groups (Diptera, Hymenoptera, Neuroptera), as well as in in vitro cultures of trypanosomatids [30,31,33,34,35,36]. Notably, in *T. theileri*-like trypanosomes, the filamentous matrix contacts only the surface of the flagellates and never the host’s intestinal cuticle. In contrast, the monoxenous flagellate *Crithidia versiformis* utilizes a similar substance to attach individual cells and conglomerates to the foregut cuticle of the green lacewing *Chrysoperla carnea* [35]. The Tthα micropopulation in the horse fly ileum includes both cells attached by flagella to the cuticle and cells at various stages of metacyclogenesis. Most of the latter lack free flagella and, without the filamentous matrix, would inevitably be expelled during defecation. In many trypanosomatids utilizing contaminative transmission, dissemination of infective stages into the environment represents the conventional route of transmission. This is common in monoxenous trypanosomatids [37] but also known in several *Trypanosoma* species, such as *T. cruzi* [1]. However, the metacyclic stages of *T. theileri*-like trypanosomes appear poorly adapted for prolonged survival in the external environment, and their primary survival strategy is to remain within the vector’s gut. The accumulation of infectious stages in the vector’s gut is not a dead-end in the parasite life cycle but rather a temporary depot. *T. theileri*-like trypanosomes typically infect their vertebrate hosts via the oral mucosa, often during the host’s defensive reactions against blood-sucking insects [2,38]—either by a strike of the tongue or by knocking the insects to the ground (e.g., using the limbs, tail, or muzzle), followed by accidental ingestion of the fallen insects together with forage plants. When the insects are crushed during mastication, a massive number of metacyclic trypanosomes are released from this depot directly onto the host’s mucosa, thereby successfully reaching their target. Thus, based on the available, mainly morphological data, the filamentous matrix performs a retaining function, preventing the unproductive excretion of parasites, primarily metacyclic forms, into the external environment. However, the filamentous matrix alone is insufficient to retain the developing parasite conglomerate within the gut, since it does not interact directly with the intestinal wall [6,31]. This anchoring function is performed by haptomonad cells capable of attaching to the cuticle via modified flagella while also being integrated into the filamentous matrix. Our data show that in Tthα trypanosomes, such cells comprise two morphotypes: epimastigotes and trypomastigotes, both randomly distributed within the matrix. Cells adjacent to the cuticular lining use a highly expanded extracellular portion of the modified undulipodium for attachment. The intraflagellar matrix of these haptomonads is saturated with filaments, and zonal hemidesmosomes form at the contact points between the flagellar membrane and the host cuticle. This attachment method is universal for trypanosomatids developing in cuticularized sections of the insect digestive system [37]. Some specific differences exist among *T. theileri*-like trypanosomes. For instance, in the hindgut of the sheep ked *M. ovinus*, the flagella of adjacent *T. melophagium* cells interact with each other by forming desmosome-like structures [9], whereas such interflagellar contacts are not observed in Tthα trypanosomes from horse flies [6]. Here, we provide the first detailed description of the attachment of Tthα flagellates to the horse fly ileum cuticle via long undulipodia. This method is characteristic of cells spatially distant from the cuticle and is observed in both epimastigotes and trypomastigotes. Attachment occurs via an expanded flagellar tip, where the membrane forms a hemidesmosome. The main part of the long undulipodium maintains its shape but lacks an axoneme and a paraxial rod. The axonemal microtubules terminate at the point where the flagellum exits the flagellar pocket. The entire intraflagellar matrix of the undulipodium is filled with longitudinally oriented filaments. A similar pattern has been described during the formation of *L. mexicana* haptomonads in vitro [39]. The diameter of intraflagellar filaments in *Leishmania* is 8–10 nm, corresponding to intermediate or septin filaments in other eukaryotes [40,41]. However, the *Leishmania* genome lacks orthologs for intermediate or septin filament proteins, such as keratins or septins [39]. In contrast, our data show that in Tthα trypanosomes, the intraflagellar filaments have a diameter of 4–6 nm, comparable to actin microfilaments. Despite progress in understanding actin and its regulators in trypanosomatid cells, its involvement in flagellar remodeling has not been previously established [42,43]. Recently, it was shown that the intraflagellar filament network in *Leishmania* haptomonads, extending from the adhesive plaque to the FAZ in the flagellar pocket, plays a crucial role in flagellar adhesion to the substrate [44]. These filaments are part of a complex system involving FAZ proteins and Kinetoplastid-Insect Adhesion Proteins (KIAPs) [45]. Specifically, KIAP2 localizes to the intraflagellar filaments of *Leishmania* haptomonads and is required for their assembly [44]. Interestingly, in the monoxenous species *Lotmaria passim*, *Crithidia mellificae*, and *C. bombi* from Hymenoptera, haptomonad flagella also possess a developed filament network linking the hemidesmosome to desmosome-like structures of the FAZ [36]. We have also described the formation of additional filament bundles associated with the FAZ on the cytoplasmic side of the plasmalemma in Tthα trypanosomes. Similar filament bundles extending deep into the cytoplasm from desmosome-like FAZ elements have previously been described in the monoxenous *Crithidia versiformis*, a parasite of the green lacewing *Chrysoperla carnea* (Neuroptera) and in *Wallacemonas raviniae* from the horse fly *Hybomitra solstitialis* [35,46]. Although the role of these cytoskeletal elements remains unclear, the fact that they are found in haptomonads representing distinct phylogenetic lineages of trypanosomatids, and therefore appear to have been conserved despite the action of diverse evolutionary factors, suggests their potential involvement in fundamental processes of cellular differentiation.

### 4.2. Ultrastructure of T. theileri-like Trypanosomes in the Vector

Until now, insights into the cellular organization of *T. theileri*-like trypanosomes have been extremely limited. Most studies have been conducted on in vitro cultures, as hemoculturing is the traditional tool for detecting infections in vertebrate hosts. Cultivation on various media is also used to describe new species and identify isolates from vectors [12,13,47,48]. In most of these works, ultrastructural analysis is of secondary importance and is generally limited to visualizing key features: the nucleus, kinetoplast, flagellar pocket, and, less frequently, other organelles [4,49,50]. Conversely, the few studies on the development of *T. theileri*-like trypanosomes in vectors previously focused primarily on host–parasite relationships, providing only fragmentary characterizations of cellular fine structure [6,9,31]. Here, we provide the first detailed investigation of the ultrastructural organization of *T. theileri*-like stages in the vector and demonstrate that the cellular organization of “wild” and “cultured” Tthα forms differs in several significant respects.

One of the key findings of our study is the discovery of a well-developed cytostome–cytopharyngeal complex (CCP) in Tthα epimastigotes. Previous ultrastructural studies on *T. theileri*-like cultures, including Tthα, failed to detect a CCP [4,12,49,50]. In dixenous trypanosomatids, two main endocytic pathways are used for macromolecule uptake: endocytosis via the flagellar pocket membrane (typical for *T. brucei* and *Leishmania* spp.) or via a specialized CCP structure, which is characteristic of most non-salivarian trypanosomes [51].

The trypanosomatid CCP includes a specialized membrane domain divided into the cytostome proper and the cytopharynx, along with associated cytostomal microtubules [52]. Since its first description in trypanosomes parasitizing fish and amphibians [52,53], this structure has subsequently been reported in the vast majority of non-salivarian species [54,55,56,57,58,59,60,61,62,63,64], with *T. theileri*-like and avian trypanosomes being the notable exceptions until now. Our study shows that in Tthα trypanosomes, the CCP is present only at the epimastigote stage attached to the ileum cuticle. To some extent, this was not unexpected. In *Trypanosoma cruzi*, where the CCP has been studied extensively using high-resolution electron microscopy, its formation and function are known to be dynamic across different life cycle stages [65,66,67]. In *T. cruzi*, the CCP is present in epimastigotes and intracellular amastigotes but absent in trypomastigotes and extracellular amastigotes, with resorption occurring during late metacyclogenesis [68]. Our data suggest that Tthα trypanosomes undergo similar processes associated with metacyclogenesis, which appears to represent the principal developmental trajectory in the host ileum: only epimastigotes possessed a CCP, while trypomastigotes (including intermediate forms and mature metacyclics) lacked this compartment. A surprising finding was the presence in the cytoplasm of *T. theileri*-like trypanosomes of coated vesicle populations oriented along the proximal part of the cytostomal microtubule bundle. Additionally, numerous smooth vesicles form along the cytostomal microtubules at the level of the cytopharynx. Generally, two main types of vesicular endocytosis are recognized in trypanosomes [51]: clathrin-coated vesicles budding from the flagellar pocket (as in *T. brucei*) [69] and smooth vesicles budding from the cytopharyngeal membrane (as in *T. cruzi*) [54,65]. However, the diversity of endocytic pathways may not be limited to these two canonical models. For example, *Trypanosoma raiae* from the blood of the ray *Raia clavata* exhibits a hybrid mode of endocytosis, possessing both a developed CCP, as in *T. cruzi*, and the ability to form coated vesicles budding from the flagellar pocket membrane, as in *T. brucei* [52].

Notably, many monoxenous trypanosomatids use a so-called “reduced cytostomal complex”, first described in *Crithidia fasciculata* [70], a parasite of culicids. This complex lacks a cytopharynx, and the cytostome always opens into the flagellar pocket [71]. Nevertheless, true CCPs with developed cytopharynges have been found in certain monoxenous genera such as *Herpetomonas*, *Paratrypanosoma*, and *Wallacemonas* [46,72,73]. Clearly, the presence of a CCP is a plesiomorphic trait inherited from free-living phagotrophic kinetoplastid ancestors [71,73,74]. Further investigation into the diversity and morphofunctional transformations of the CCP remains a promising direction for future research.

Previously, many authors reported the presence of unusually high amounts of large acidocalcisomes in the cytoplasm of cultured *T. theileri*-like trypanosomes [4,49,50]. Interestingly, in *Trypanosoma cyclops*, a flagellate parasite of primates phylogenetically related to *T. theileri*-like trypanosomes [75], the cytoplasm of cultured epimastigotes is also densely packed with acidocalcisomes (“pigment bodies”, sensu [55]). A 3D reconstruction analysis based on serial ultrathin sections previously performed by our group [12] showed that elongated epimastigotes of cultured Tthα trypanosomes may contain 100 or more acidocalcisomes per cell. In contrast, our results demonstrate that epimastigotes and metacyclic trypomastigotes of Tthα in the vector contain only small numbers of these organelles. A similar pattern was observed for glycosomes: their number may reach 500 or more per cell in elongated Tthα epimastigotes and approximately 50 in pyriform epimastigotes in culture [12], whereas in the vector their abundance is low and comparable to that of acidocalcisomes [6]. Interestingly, other authors did not report substantial numbers of glycosomes in cultured *T. theileri*-like trypanosomes [49,50]. Acidocalcisomes are known to play important roles in osmoregulation, autophagy, and resistance to environmental stress throughout the parasite life cycle [76]. Glycosomes are also essential components of trypanosomatid metabolism, containing numerous metabolic enzymes, most notably those involved in glycolysis [77,78]. In bloodstream stages of African *Trypanosoma brucei*, which rely exclusively on glycolysis for energy production, the presence of large numbers of glycosomes (>60 per cell) appears entirely expected [79]. In these trypanosomes, glycosome numbers increase immediately after the onset of the cell cycle and are subsequently maintained at a high level. It has been suggested that the large number of glycosomes in bloodstream forms of *T. brucei* is associated with increasing energetic demands as cell size increases [80]. Since metacyclic *T. theileri*-like trypanosomes rapidly form populations of large, motile epimastigotes after inoculation into culture medium [12], we suggest that the sharp increase in glycosome and acidocalcisome numbers in cultured stages may likewise be associated with processes of cell growth. Another important feature of the “wild” *T. theileri*-like forms from the vector described in the present study is the presence of a single large lysosome-like vacuole in the cytoplasm, which was not detected in cultured cells of this species [12]. Any hypotheses regarding the nature and possible functions of this structure are based solely on the interpretation of ultrastructural data. This vacuole, bounded by a single membrane, is morphologically most similar to megasomes, the large lysosomal organelles of *Leishmania* amastigotes [81,82]. Vacuoles containing a homogeneous fine-grained matrix (Figure 3F) also resemble certain forms of *T. cruzi* reservosomes [83,84]. The presence of numerous membrane fragments within the lysosome-like vacuoles of *T. theileri*-like trypanosomes may indicate their involvement in autophagic processes, which are known to play an important role in cellular remodeling in many trypanosomatids, including during metacyclogenesis [85].

## 5. Conclusions

In this study, we demonstrated that the final phase of *T. theileri*-like trypanosome development in the horse fly *H. montana* is spatially restricted to the host’s ileum. This is facilitated by a dense fibrillar matrix in which the trypanosome cells are embedded. The resulting parasite conglomerate is retained near the gut wall by cells attached to the surface of the ileum epicuticle via modified flagella. The primary distinguishing feature of these flagella is the absence of an axoneme and a paraxial rod in their extracellular portion, along with an intraflagellar matrix saturated with 4–6 nm filaments that morphologically connect the hemidesmosome of the attachment plaque with the FAZ (Flagellar Attachment Zone).

The heteromorphic trypanosome micropopulation in the horse fly ileum consists of two morphotypes: epimastigotes and trypomastigotes. We documented several fundamental differences in their cellular organization. For the first time among the *T. theileri*-like trypanosomes, a complete cytostome–cytopharyngeal complex opening independently of the flagellar pocket was identified in epimastigotes. Furthermore, a single large lysosome-like vacuole forms at the posterior end of epimastigote cells, whose contents may be released through perforation of the plasma membrane. We showed that, unlike epimastigotes, *T. theileri*-like trypomastigotes lack not only the cytostome–cytopharyngeal complex but also most elements of the secretory-transport system. During metacyclogenesis—the successive stages of which, unfortunately, could not be traced in this study—the parasite cells undergo progressive simplification and compaction.

A comparison of our findings with previous ultrastructural studies of *T. theileri*-like trypanosomes conducted on hemocultures revealed that the stages displaying the most complete set of ultrastructural features are absent among cultured forms. This problem might potentially be resolved through further refinement of cultivation methods or, if such methods cannot be improved, through more detailed investigations of a broad range of trypanosomes in their natural vectors.

## Data Availability

Data used in this article can be found in the main text, or (in the case of sequences) in the GenBank database (see the list of accession numbers in Section 2).
